# Supplementary material for: Sociodemographic Factors Associated with COVID-19 Vaccination among People in Guatemalan Municipalities
Source: Vaccines (Basel). 2023 Mar 28;11(4):745. doi: 10.3390/vaccines11040745 (PMC10143355; doi:10.3390/vaccines11040745)
Supplement: Supplementary file 1 [file vaccines-11-00745-s001.zip › vaccines-2296998-supplementary.pdf]

Supplemental Table S1. Association between sociodemographic factors (by municipalities and departments) and two-dose vaccination coverage (%) by municipalities in Guatemala (N = 336). SARS-CoV-2 case and vaccination data are from February 13, 2020 to October 1, 2021.

	BIVARIATE MODEL					FULL MULTIVARIABLE MODEL					SIMPLIFIED MULTIVARIABLE MODEL				
	Coefficient	95% Confidence Interval		Marginal R^2	Conditional R^2	Coefficient	95% Confidence Interval		Marginal R^2	Conditional R^2	Coefficient	95% Confidence Interval		Marginal R^2	Conditional R^2
Null multi-level model	43.766	39.645	47.949	0.000	0.239										
Municipal level variables															
% Mayan	-0.151	-0.206	-0.095	0.120	0.342	0.026	-0.028	0.081			-	-	-		
% Rural residence	-0.188	-0.251	-0.127	0.111	0.246	-0.001	-0.067	0.062			-	-	-		
% Educational level primary school or above	1.400	1.184	1.616	0.400	0.451	1.046	0.714	1.386			0.877	0.613	1.138		
% Female sex	2.527	1.022	4.026	0.036	0.281	1.793	0.398	3.169			2.154	0.959	3.354		
% in 0 – 17 age group	-1.813	-2.158	-1.470	0.296	0.388	0.599	-0.015	1.218			-	-	-		
% in 60 or older age group	4.538	3.566	5.485	0.218	0.412	2.369	1.114	3.678			1.628	0.656	2.571		
% tested for SARS-CoV-2	0.521	0.431	0.613	0.314	0.460	0.242	0.112	0.370	0.512	0.583	0.253	0.154	0.356	0.494	0.560
% died due to COVID-19	1.171	0.939	1.406	0.249	0.406	0.188	-0.124	0.491			-	-	-		
Departmental level variables															
Under-5 childhood mortality rate	-0.087	-0.461	0.268	0.003	0.249	0.188	-0.064	0.431			-	-	-		
% reporting difficulty accessing healthcare facility due to distance	-0.822	-1.121	-0.532	0.176	0.249	-0.043	-0.466	0.377			-	-	-		
% 12-23 month olds receiving third	0.653	0.201	1.106	0.073	0.244	0.110	-0.301	0.522			-	-	-		

Pentavalent vaccine											
Gini coefficient	-0.619	-1.195	-0.084	0.052	0.245	-0.201	-0.682	0.270	-	-	-
